# Supplementary material for: Towards layer-selective quantum spin hall channels in weak topological insulator Bi4Br2I2
Source: Nat Commun. 2023 Aug 16;14:4964. doi: 10.1038/s41467-023-40735-7 (PMC10432521; doi:10.1038/s41467-023-40735-7)
Supplement: Supplementary file 1 — Supplementary Information [file 41467_2023_40735_MOESM1_ESM.pdf]

Supplementary Information for

Towards Layer-Selective Quantum Spin Hall Channels in Weak Topological Insulator $Bi_4Br_2I_2$

Jingyuan Zhong[1], Ming Yang[1], Zhijian Shi[1], Yaqi Li[1], Dan Mu[2], Yundan Liu[2], Ningyan Cheng[3], Wenxuan Zhao[4], Weichang Hao[1,5], Jianfeng Wang[1,*], Lexian Yang[4,*], Jincheng Zhuang[1,5,*], Yi Du[1,5,*]

[1] School of Physics, Beihang University, Haidian District, Beijing 100191, China

[2] Hunan Key Laboratory of Micro-Nano Energy Materials and Devices, and School of Physics and Optoelectronics, Xiangtan University, Hunan 411105, China

[3] Information Materials and Intelligent Sensing Laboratory of Anhui Province, Key Laboratory of Structure and Functional Regulation of Hybrid Materials of Ministry of Education, Institutes of Physical Science and Information Technology, Anhui University, Hefei, Anhui, China

[4] State Key Laboratory of Low Dimensional Quantum Physics, Department of Physics, Tsinghua University, Beijing 100084, China

[5] Centre of Quantum and Matter Sciences, International Research Institute for Multidisciplinary Science, Beihang University, Beijing 100191, China

Jingyuan Zhong, Ming Yang, and Zhijian Shi contributed equally to this work.

*Correspondence authors. E-mail: jincheng@buaa.edu.cn; lxyang@tsinghua.edu.cn; wangjf06@buaa.edu.cn; yi_du@buaa.edu.cn

Contents

1. Mole ratio in $Bi_4Br_2I_2$ (Supplementary Note 1, Figure 1).

2. Identification of Br and I atoms by STM images (Supplementary Note 2, Figure 2).

3. Different stacking modes of α-Bi_4I_4, α'-Bi_4Br_4, and $Bi_4Br_2I_2$ (Supplementary Note 3, Figure 3).

4. Monolayer of $Bi_4Br_2I_2$ as the QSH insulator (Supplementary Note 4, Figure 4).

5. WTI nature of 3D $Bi_4Br_2I_2$ (Supplementary Note 5, Figure 5-9).

6. ARPES spectra of cleaved (001) surface by Helium light (~21.2 eV) (Supplementary Note 6, Figure 10).

7. Fitting details of EDC at (100) surface (Supplementary Note 7, Table 1-4).

8. Detailed calculations of the energy gaps (Supplementary Note 8, Figure 11).

9. Layer-selective QSH channels by changing chemical potential (Supplementary Note 9, Figure 12-15).

10. Hamiltonian of TSS of $Bi_4Br_2I_2$ (Supplementary Note 10, Figure 16).

11. Discussion about virtual crystal approximation (Supplementary Note 11).

Supplementary Note 1. Mole ratio in $Bi_4Br_2I_2$.

For characterizing the mole ratio of Bi, Br, and I, energy dispersive spectroscopy measurement is conducted as shown in Supplementary Figure 1. The mole ratio of Bi, Br, and I elements are 48.76%, 25.22%, and 26.02, respectively, indicating the approximate 1:1 mole ratio of Br and I atoms in our sample.

Supplementary Note 2. Identification of Br and I atoms by STM images.

Supplementary Figure 2 displays the STM images of α-Bi_4I_4, α'-Bi_4Br_4, and $Bi_4Br_2I_2$ to make a comparative study of the surface topography of these three samples. The enlarged STM topography of (001) surface of α-Bi_4I_4, α'-Bi_4Br_4, and $Bi_4Br_2I_2$ are shown in Supplementary Figure 2d-f, respectively, where the highly quasi-1D features of these three compounds can be clearly distinguished. The atomic structural models are projected onto the topography with the high consistency. For α-Bi_4I_4 and α'-Bi_4Br_4, the uniform distribution of surface atoms is observed due to the single kind of halogen atoms. However, the STM images of $Bi_4Br_2I_2$ displays two kinds of atoms, resulting from the substitution of Br by I. The different radius of Br and I ions lead to the dark and light protrusions randomly distributed in the location of halogen atoms. The STM images imply the successful fabrication of $Bi_4Br_2I_2$ single crystal.

Supplementary Note 3. Different stacking modes of α-Bi_4I_4, α'-Bi_4Br_4, and $Bi_4Br_2I_2$.

The high-angle annular dark-field scanning transmission electron microscopy (HAADF-STEM) results of (010) surface of α-Bi_4I_4, α'-Bi_4Br_4, and $Bi_4Br_2I_2$ are displayed in Supplementary Figure 3a-c, respectively, where the double-layer and triple-layer unit cells can be directly distinguished[1-5]. Clearly, the α-Bi_4I_4 shows unidirectional stacking mode with double-layer unit cell. On the contrary, the 180-degree rotation of adjacent layers compared to α-Bi_4I_4 is identified in α'-Bi_4Br_4 result. The $Bi_4Br_2I_2$ shows the combined stacking mode of α-Bi_4I_4 and α'-Bi_4Br_4, where the triple-layer unit cell including two unidirectional layers plus one antiparallel layer alternatively stacking along *c* axis, namely the A_1-A_2-B stacking mode.

With the EDS mapping results, the concentrated location of Bi, Br, and I atoms can be spatially distinguished as shown in Supplementary Figure 3d-f. For the Bi content, the light areas are evenly

distributed among different layers, as shown in Supplementary Figure 3d, indicating the identical concentration of Bi atoms along inter-chain direction. Each layer has higher intensity at the middle position of every chain, corresponding to the positions of Bi atoms in the crystal structure. Supplementary Figure 3f exhibits the distribution of I atoms, which locates at the interlayer space of two unidirectional layers, A_1 and A_2. The two unidirectional layers are similar with the unit cell of α-Bi_4I_4, which is the stable product with only I as the halogen atoms. To sum up, with the comparison among different stacking modes observed by HAADF-STEM and EDS mapping results, the triple-layer structure of synthesized $Bi_4Br_2I_2$ is firmly evidenced.

Supplementary Note 4. Monolayer of $Bi_4Br_2I_2$ as the QSH insulator.

The band structure of (001) monolayer of $Bi_4Br_2I_2$ is calculated, as shown in Supplementary Figure 4. The band inversion with different parities at M point is observed after turn on the SOC, as displayed in Supplementary Figure 4a-b, where the direct gap and indirect gap are approximately 341 meV and 297 meV, respectively. Supplementary Figure 4c displays the band structure of $Bi_4Br_2I_2$ monolayer nanoribbon by Green function methods, where Dirac cone features could be clearly seen. Based on the evolutions of Wannier function centers in Brillion zone, we have calculated the $\mathbb{Z}_2$ topological invariant within the first-principle framework, where the $\mathbb{Z}_2 = 1$, as shown in Supplementary Figure 4d. All these results demonstrate the QSH nature of monolayer $Bi_4Br_2I_2$.

Supplementary Note 5. WTI nature of 3D $Bi_4Br_2I_2$.

The WTI nature of triple-layer $Bi_4Br_2I_2$ can be characterized by four $\mathbb{Z}_2$ invariants calculated by the evolution of WCCs in different planes[6-7]. The six calculated planes in bulk BZ are shown in Supplementary Figure 5, where the $k_x = 0$, $k_x = \pi$, $k_y = 0$, and $k_y = \pi$ planes shown neatly parallel evolution paths with zero intersecting points, implying $\mathbb{Z}_2 = 0$ (0 mod 2 equals to 0). However, the WCCs evolution of $k_z = 0$ and $k_z = \pi$ planes exhibit three intersecting points, implying the topological non-trivial $\mathbb{Z}_2 = 1$ (3 mod 2 equals to 1). Considering all six planes with six intersecting points, the topological phases can be characterized as WTI (6 mod 2 equals to 0). To sum up, the four $\mathbb{Z}_2$ topological invariants are calculated as (0; 0 0 1), indicating the WTI nature of 3D $Bi_4Br_2I_2$.

The calculation according to Green function methods exhibits similar results with DFT methods, as displayed in Supplementary Figure 6. The band structure of the projected (001) plane possesses three

pairs of inverted bands with ~230 meV gap in Supplementary Figure 6a, while the (100) plane has gapless topological surface states with complicated coupling behavior as indicated in Supplementary Figure 6b-d. For further clarification of (100) topological surface states, the constant energy contours (CECs) at different are displayed in Supplementary Figure 6e-h. The CECs at 0 meV and -47 meV exhibit highly anisotropic characteristic, where three pairs of quasi-linear lines are parallel with k_z direction, implying the barely zero dispersion. On the contrary, the Dirac-type energy momentum dispersion along k_y direction indicates the large group velocity along chain direction. At deeper energy level, the type of carriers transforms into hole-type from electron-type.

In our calculations, the energy cutoff of 300 eV is used, and 9 × 9 × 1 and 13×13×2 Γ-centered k-grid meshes are adopted for structural relaxation and electronic structure calculations, respectively.

To demonstrate the computational accuracy, we conducted energy convergence tests on the energy cutoff and k-grid mesh. As shown in Supplementary Figure 7, the total energy of a unit cell for $Bi_4Br_2I_2$ converge when the energy cutoff is larger than 250 eV, and the k-grid mesh along the a^* and b^* is denser than 5 × 5; while the energy convergence is achieved even the number of c^* grid points is 1. All these tests indicate that the parameter settings in our work are reasonable.

In addition, the tight-binding (TB) Hamiltonian based on the maximally localized Wannier functions (MLWF) is used. In Supplementary Figure 8, we compare the band structures calculated by TB model (red dashed lines) and first-principles (black solid lines). The good matching between them demonstrates that a well fitted MLWF is achieved. The calculated band structures of $Bi_4Br_2I_2$ with and without SOC along the full high-symmetry lines[8-9] are shown in Supplementary Figure 9.

Supplementary Note 6. ARPES spectra of cleaved (001) surface by Helium light (~21.2 eV).

Due to the Lifshitz transition in this system, that the Fermi level moves upward with the decrement of temperature. Thus, the ARPES measurement (Helium light ~21.2 eV) measured at 6 K is performed to exhibit more information of conduction bands. The CECs of (001) surface at different binding energies are shown in Supplementary Figure 10a, where both of the conduction band and valence band could be seen clearly. The energy momentum dispersion along chain direction at $\bar{M}$ and $\bar{\Gamma}$ points are shown in Supplementary Figure 10b-c, respectively, where similar results with laser ARPES in the main text are observed. The gap size along $\bar{M}$ direction can be calculated ~100 meV in the MDC spectra in Supplementary Figure 10d, consisting with the laser ARPES and STS results in the main

text. With the combination of He light ARPES, laser ARPES and STS spectra, the gap size of (001) inverted bulk band can be rationally characterized as ~100 meV.

Supplementary Note 7. Fitting details of EDC at (100) surface.

In order to figure the detailed information of ARPES results, we performed the fitting of EDC curves of (100) surface. The EDC of (100) topological surface states in $Bi_4Br_2I_2$ is fitted by multiple Lorentz-type peaks in the main text Fig. 3g and 3i, which are described by the Lorentz-type distribution equation[10-11]:

$$y = y_0 + \frac{2A}{\pi}\left[\frac{w}{4(x-x_c)^2+w^2}\right] \qquad \text{(Supplementary Equation 1)}$$

where $A = \frac{(y_c-y_0)}{2}w\pi$, y_0 represents the background signal, (x_c, y_c) is the coordinates of peak maximum, w is the full with at half maximum. Each Lorentz peak can be fully described by three independent parameters x_c, y_c, and w. Noticing that the value of EDC peak maximum is meaningless, only the relatively value of y_c of different peaks in the same EDC counts. The fitting parameters of corresponding to Fig. 3, x_c, y_c, and w, are listed in Supplementary Table 1-4. Similar processes are conducted to gain the energy location of topological surface states in different EDCs, which are used to label the band structure dispersions in the main text. Interestingly, the outer topological surface states show higher intensity, which can be separated into two pairs of quasi-linear bands by step-by-step fitting, consisting with the two nearby outer topological surface states calculated by simulation results.

Supplementary Note 8. Detailed calculations of the energy gaps.

The calculated topological surface states of (100) plane with denser k points are shown in Supplementary Figure 11. The topological surface states along $\bar{G}-\bar{X}$ direction are displayed in Supplementary Figure 11a-b, where each two of the three pairs of topological surface states couple to open a finite gap. The much smaller gaps formed by A_1-B coupling and A_1-A_2 coupling than A_2-B coupling imply the weaker coupling strength between A_1-B layers and A_1-A_2 layers.

The multiple perspectives of simulation rationally confirm the complex coupling behavior of three pairs of topological surface states of (100) surface $Bi_4Br_2I_2$, showing high consistency with the experimental results measured by laser ARPES. Thus, we believe the triple-layer WTI $Bi_4Br_2I_2$

possesses the potential to achieve the layer-selective quantum spin Hall conducting channels by adjusting the Fermi level to locate at certain gap induced by interlayer coupling.

Supplementary Note 9. Layer-selective QSH channels by changing chemical potential.

For the WTI constituted by single-layer, the topological surface states are protected by the translational symmetry and time-reversal symmetry, exhibiting the identical characteristics in all layers. However, the triple-layer WTI $Bi_4Br_2I_2$ possesses three distinguished layers, making it possible to achieve unique topological surface states. The coupling between each two of the triple-layer unit cell contributes three different coupling gaps, providing the opportunities to tune the on/off states of QSH channels in different layers. The Fig. 4 in the main text exhibits three kinds of conducting channels, where the Supplementary Figure 12 displays other two sets of conducting channels at -16 meV and -20 meV, representing the B-layer solely conducting situation and A_1-A_2-layer combined conducting situation, respectively.

Next, we demonstrate that the charge carriers doping can efficiently tune the Fermi level to different binding energies. In calculations, we simulate the electron or hole doping in the system by adding or reducing additional electrons. As shown in Supplementary Figure 13, we compare the calculated surface band structures with no and 0.1 extra electrons added into the 11-layers-thick (100) slab. It can be seen that the charge doping can indeed cause the rigid shift of the Fermi level with the energy bands almost unchanged (especially for the surface state bands), and 0.1 electrons doping can induce a Fermi energy shift of almost the same energy value (0.106 eV). Hence, it is feasible to tune the Fermi surface at different gap regions simply by electric gating method or charge carriers doping. In the following we show the first-principles Fermi surfaces just by tuning the Fermi level to some certain binding energies.

As plotted in Supplementary Figure 14, for most Fermi energies from -0.1 eV to 0.1 eV, there are six bands with weak k_z dispersion in the whole Brillouin zone (or three bands in the half Brillouin zone of k_y>0), corresponding to three pairs of helical edge states, e.g., at the binding energy of E_1, E_4, and E_5. While for the Fermi energy at E_2 or E_3, where a coupling gap is opened between two edge states, only one pair of helical edge states remains. The spin texture information is also plotted in Supplementary Figure 14, consistent with the results shown in our manuscript. Consequently, as schematically plotted in Fig. 4h, the non-degeneracy of the three channels and the interlayer interaction-induced the energy

gap provide the additional degree of freedom to control the QSH channels in selective layers in $Bi_4Br_2I_2$ by charge carriers doping or electric gating method.

Here, we also calculate the partial (band decomposed) charge densities with a very small energy range around the selected binding energies in the entire half Brillouin zone instead of only along Γ-X. As shown in Supplementary Figure 15, the charge distributions for Fermi energy at E_1 and E_2 are almost the same as those in Fig. 4e and 4f, respectively, demonstrating the validity of the charge distribution in Fig. 4e-g. Since the energy window for E_3 is very small, a denser k-grid is needed, requiring expensive computational burden, so we did not obtain the result of partial charge density around E_3 for comparison with Fig. 4g. The yellow color in Supplementary Figure 15b-c (also in Fig. 4e-g of manuscript) indicates the positive group velocities with an up spin for the surface states in the half Brillouin zone of $k_y > 0$; while their time-reversal partners in the half Brillouin zone of $k_y < 0$ will carry the negative group velocities with a down spin. Due to the different charge distributions at different binding energies in Fig. 4e-g, layer-selective QSH channels can be controlled by gating or charge carrier doping, as schematically shown in Fig. 4h of the main text.

Supplementary Note 10. Hamiltonian of TSS of $Bi_4Br_2I_2$.

Here we start from the edge state Hamiltonian of QSH, and introduce the interlayer coupling on the (100) surface to build the surface state models, which are then discussed to compare with the DFT or ARPES results.

The Hamiltonian of edge states of a QSH can be written as

$$H_0 = \hbar v_F k_y \sigma_z, \qquad \text{(Supplementary Equation 2)}$$

where we have supposed the edge state channel is along the y direction, consistent with the chain direction of Bi_4X_4 or $Bi_4Br_2I_2$, and σ_z denotes the spin degree of freedom. Such helical spin-polarized bands are shown in Supplementary Figure 16a.

The bulk material is accumulated by QSH insulators with different stacking orders with weak vdW force. For β-Bi_4I_4, only a single Bi_4I_4 block is arranged along lattice c axis, engendering the formation of WTI phase. The (100) surface of β-Bi_4I_4 remains all the QSH edge states. Here and henceforth, ignoring the k_z dispersion, its surface state Hamiltonian is the same as that of QSH edge states, i.e., $H_1 = H_0 = \hbar v_F k_y \sigma_z$.

For α-Bi_4I_4 and α'-Bi_4Br_4, there are two blocks with layer glide and/or layer rotation as the unit cell,

resulting in two nondegenerate edge states. In this case, the energy gap opens at the cross points of two edges states in the reciprocal space due to the interlayer coupling. Thus, their (100) surface state Hamiltonian can be written as

$$H_2 = \hbar v_F k_y \sigma_z \tau_0 + m\sigma_y \tau_y + \varepsilon \tau_z, \qquad \text{(Supplementary Equation 3)}$$

where τ denotes the layer degree of freedom, m and ε are the coupling and onsite energy difference between two edge states from two different layers respectively. In Supplementary Equation 3, we have supposed the same Fermi velocity of these two edge states. With different Fermi velocities and asymmetric potential for two layers, the above Hamiltonian is further written as

$$H_2' = \begin{pmatrix} \hbar v_{F1} k_y + \varepsilon_1 & 0 & 0 & -m \\ 0 & -\hbar v_{F1} k_y + \varepsilon_1 & -m & 0 \\ 0 & -m & \hbar v_{F2} k_y + \varepsilon_2 & 0 \\ -m & 0 & 0 & -\hbar v_{F2} k_y + \varepsilon_2 \end{pmatrix}.$$

(Supplementary Equation 4)

The corresponding band with specific values of parameters is shown in Supplementary Figure 16b. Two helical edge states with an interlayer-coupling gap are formed on the (100) surface of α-Bi_4I_4 and α'-Bi_4Br_4, which is consistent with the DFT and ARPES results[3].

For $Bi_4Br_2I_2$, there are three blocks in a unit cell, resulting in three nondegenerate edge states. The energy gap opens at the cross points of every two edges states due to the interlayer coupling. It (100) surface state Hamiltonian can be written as

$$H_3 = \begin{pmatrix} \hbar v_{F1} k_y + \varepsilon_1 & 0 & 0 & -m_{12} & 0 & -m_{13} \\ 0 & -\hbar v_{F1} k_y + \varepsilon_1 & -m_{12} & 0 & -m_{13} & 0 \\ 0 & -m_{12} & \hbar v_{F2} k_y + \varepsilon_2 & 0 & 0 & -m_{23} \\ -m_{12} & 0 & 0 & -\hbar v_{F2} k_y + \varepsilon_2 & -m_{23} & 0 \\ 0 & -m_{13} & 0 & -m_{23} & \hbar v_{F3} k_y + \varepsilon_3 & 0 \\ -m_{13} & 0 & -m_{23} & 0 & 0 & -\hbar v_{F3} k_y + \varepsilon_3 \end{pmatrix},$$

(Supplementary Equation 5)

where m is the coupling between arbitrary two different layers (denoted by subscripts) and ε is the onsite potential of different layers. Its corresponding band with specific values of parameters is shown in Supplementary Figure 16c. Three helical edge states with interlayer-coupling gaps are formed on the (100) surface of $Bi_4Br_2I_2$, which is roughly consistent with our DFT calculations. In Supplementary Figure 16d, we fit our model to the DFT calculated band structure, and obtain the reasonable

parameters as listed in the caption of Supplementary Figure 16, where the subscripts of 1, 2 and 3 represent the A_2, B and A_1 layers respectively. Thus, our Hamiltonian of Supplementary Equation 5 can well describe the band of surface states of $Bi_4Br_2I_2$.

Supplementary Note 11. Discussion about virtual crystal approximation.

To study disordered system such as alloys and solid solutions with first-principles calculation method, approximations must be employed to take into consideration the disorder. The direct type is to build a supercell with random distribution of atoms, but such calculations generally require the use of very large supercells in order to imitate the distribution of local chemical environments, and tend to be computationally very demanding. A much simpler and computationally less expensive approach is to employ the virtual crystal approximation (VCA), in which one studies a crystal with the primitive periodicity, but composed of fictitious virtual atoms that interpolate between the behavior of the atoms in the parent compounds.

The implementation of VCA in VASP closely follows the methodology suggested by Bellaiche and Vanderbilt[12], in which the pseudopotentials of a virtual atom are mixed from the pseudopotentials of its parent atoms according to specific weight:

$$V_{\mathrm{ps}}(\mathbf{r},\mathbf{r}') = \sum_{\alpha} \omega_{\alpha} V_{\mathrm{ps}}^{\alpha}(\mathbf{r},\mathbf{r}'), \qquad \text{(Supplementary Equation 6)}$$

where, V_{ps} and V_{ps}^{α} is the pseudopotentials of virtual atom and its parent atoms respectively, ω_{α} is the weight. If the pseudopotentials of this parent atoms involved in the mixing are not vastly different, this approximation can deliver satisfactory results, e.g., in $Pb(Zr_{0.5}Ti_{0.5})O_3$, GeSn alloy, $Al_xIn_{1-x}P$[13-16].

In this work, we design a virtual atom at the Br/I site, whose pseudopotential is mixed from 0.5 weight of Br atoms and 0.5 weight of I atoms, to simulate the uniform and disordered distributions of Br/I found in experiments. Since Br and I are both VIIA group elements, separated by only one period in the periodic table, their pseudopotentials are likely to be similar, making the results of VCA reliable. Importantly, by employing the method of VCA, our calculated surface band structure matches well with the experimental ARPES measurements. As for the calculated energy gap of surface states, it should exist due to the allowed interlayer coupling, but there may be a small deviation in its size.

EDC at $\bar{\Gamma}$ point with $k_y = 0$			
Name	x_c (meV)	y_c (a.u.)	w (meV)
Bulk peak	-101.7	$5.95*10^{-5}$	80.8
TSS peak 1	-6.7	$4.25*10^{-5}$	16.5
TSS peak 2	-39.7	$2.39*10^{-4}$	43.2

Supplementary Table 1. EDC fitting parameters at $\bar{\Gamma}$ point with $k_y = 0$

EDC at $\bar{Z}$ point with $k_y = 0$			
Name	x_c (meV)	y_c (a.u.)	w (meV)
Bulk peak	-115.6	$8.36*10^{-5}$	81.2
TSS peak 1	-8.8	$8.06*10^{-5}$	16.9
TSS peak 2	-37.4	$1.48*10^{-4}$	56.2

Supplementary Table 2. EDC fitting parameters at $\bar{Z}$ point with $k_y = 0$

EDC at $\bar{\Gamma}$ point with k_y = 0.007 Å^{-1}			
Name	x_c (meV)	y_c (a.u.)	w (meV)
Bulk peak	-103.6	$5.23*10^{-5}$	50.0
TSS peak 1	-10.4	$6.40*10^{-5}$	9.5
TSS peak 2	-17.9	$9.65*10^{-5}$	12.9
TSS peak 3	-26.9	$1.33*10^{-4}$	20.6
TSS peak 4	-55.7	$1.50*10^{-4}$	42.6

Supplementary Table 3. EDC fitting parameters at $\bar{\Gamma}$ point with k_y = 0.007 Å^{-1}

EDC at $\bar{Z}$ point with k_y = 0.005 Å^{-1}			
Name	x_c (meV)	y_c (a.u.)	w (meV)
Bulk peak	-100.1	6.46*10^{-5}	59.4
TSS peak 1	-9.7	6.59*10^{-5}	12.0
TSS peak 2	-18.4	6.52*10^{-5}	15.2
TSS peak 3	-28.6	1.36*10^{-4}	23.7
TSS peak 4	-54.8	8.57*10^{-5}	43.4

Supplementary Table 4. EDC fitting parameters at $\bar{Z}$ point with k_y = 0.005 Å^{-1}

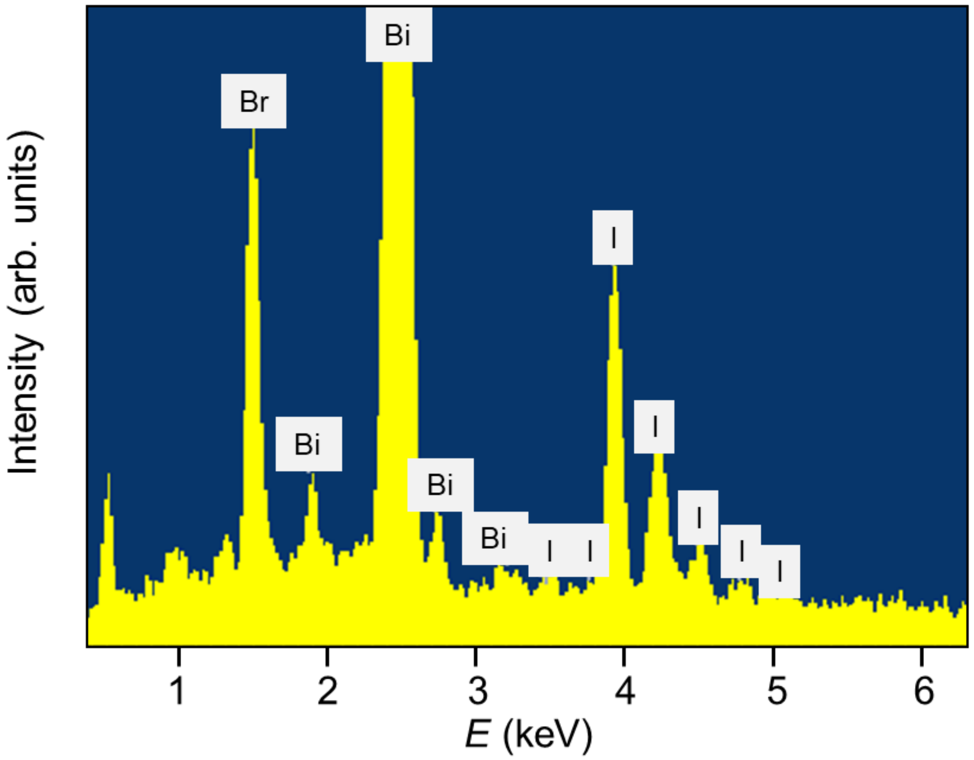

Supplementary Figure 1. EDS results. EDS results of $Bi_4Br_2I_2$ with the labelled peaks corresponding to each element. The mole ratio of Bi, Br, and I elements are 48.76%, 25.22%, and 26.02, respectively, indicating the 1:1 mole ratio of Br and I atoms.

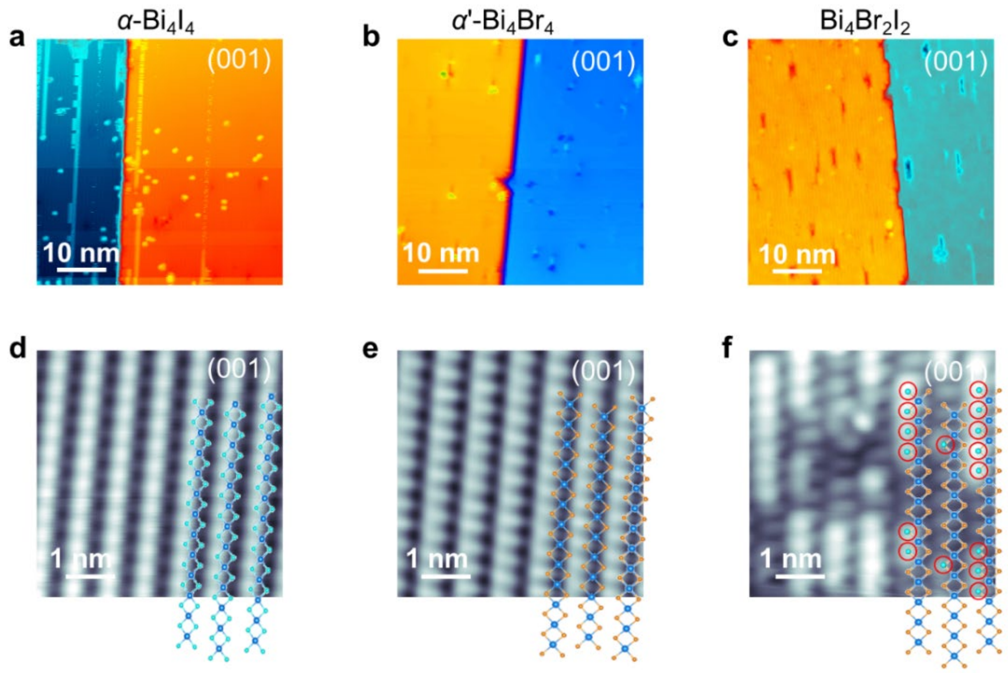

Supplementary Figure 2. Additional STM results. a-c, Large-scale STM topography of (001) plane of α-Bi_4I_4, α'-Bi_4Br_4, and $Bi_4Br_2I_2$, respectively. **d-f**, High-resolution STM topography of (001) plane in α-Bi_4I_4, α'-Bi_4Br_4, and $Bi_4Br_2I_2$, respectively. The schematics of (001) plane atoms are projected, where deep blue, light blue, and orange balls represent Bi, Br, and I, respectively. The red circles denote the lighter areas in **f**, indicating the randomly distributed halogen atoms of Br and I.

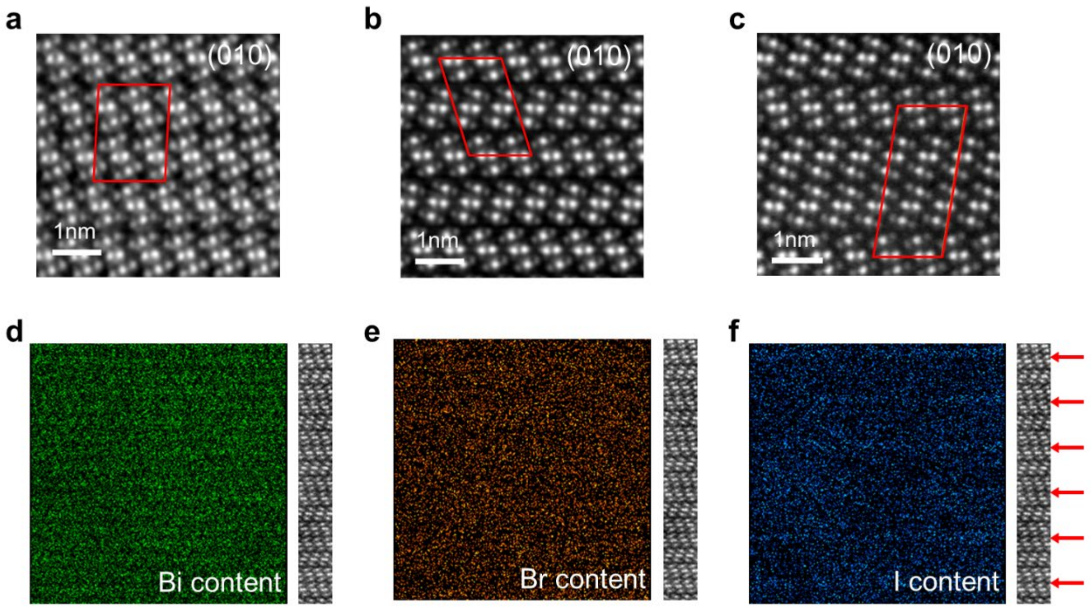

Supplementary Figure 3. Additional TEM and EDS results. a-c, HAADF-STEM results of (010) in α-Bi_4I_4, α'-Bi_4Br_4, and $Bi_4Br_2I_2$, respectively. Two double-layer unit cells and one triple-layer unit cell are labelled with red quadrangles. **d-f**, EDS mapping spectra of triple-layer $Bi_4Br_2I_2$ with Bi, Br, and I content, respectively. Each of the right panels is the corresponding HAADF-STEM image of (010) structure of $Bi_4Br_2I_2$. The red arrows indicate the concentrated location of I content.

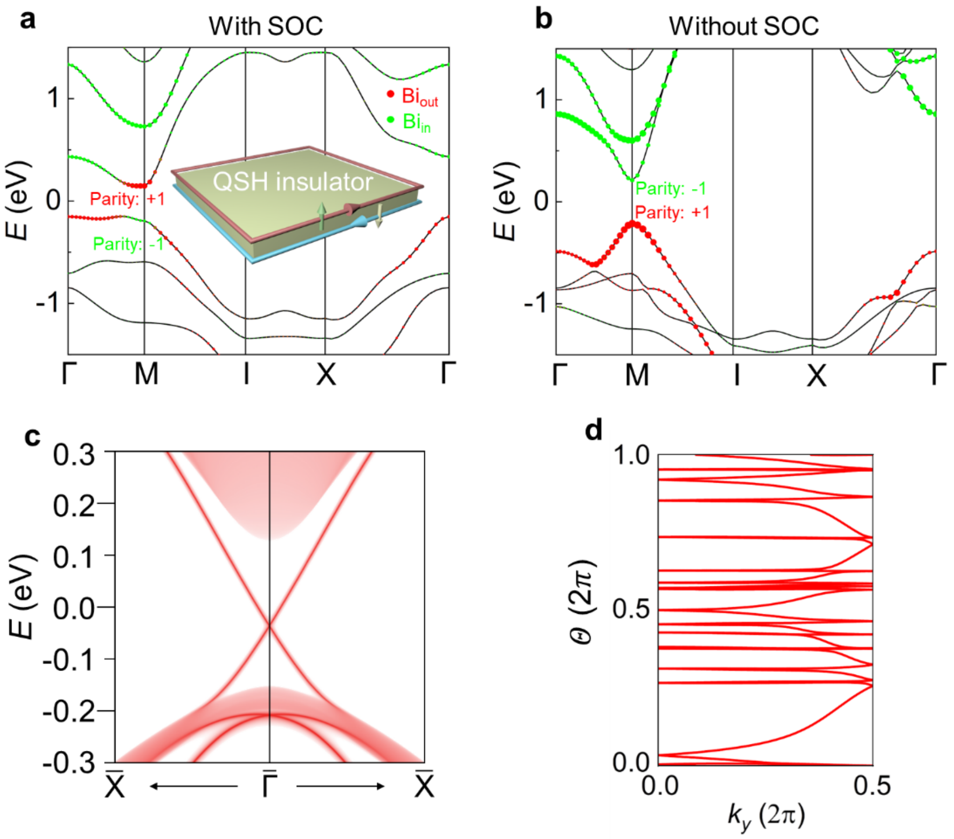

Supplementary Figure 4. Band structure of the monolayer (001) plane of $Bi_4Br_2I_2$. a, Calculated band structure of the monolayer (001) plane of $Bi_4Br_2I_2$ with SOC. The inset is plotted to indicate the QSH nature of monolayer $Bi_4Br_2I_2$. **b**, Calculated band structure of the monolayer $Bi_4Br_2I_2$ without SOC. **c,** Band structure of $Bi_4Br_2I_2$ nanoribbon, where the linear energy-momentum dispersion is observed. **d**, Evolutions of Wannier charge centers (WCCs) along k_y of monolayer $Bi_4Br_2I_2$, where each k_x intersects the red line once, indicating the nontrivial topological invariant of $\mathbb{Z}_2 = 1$.

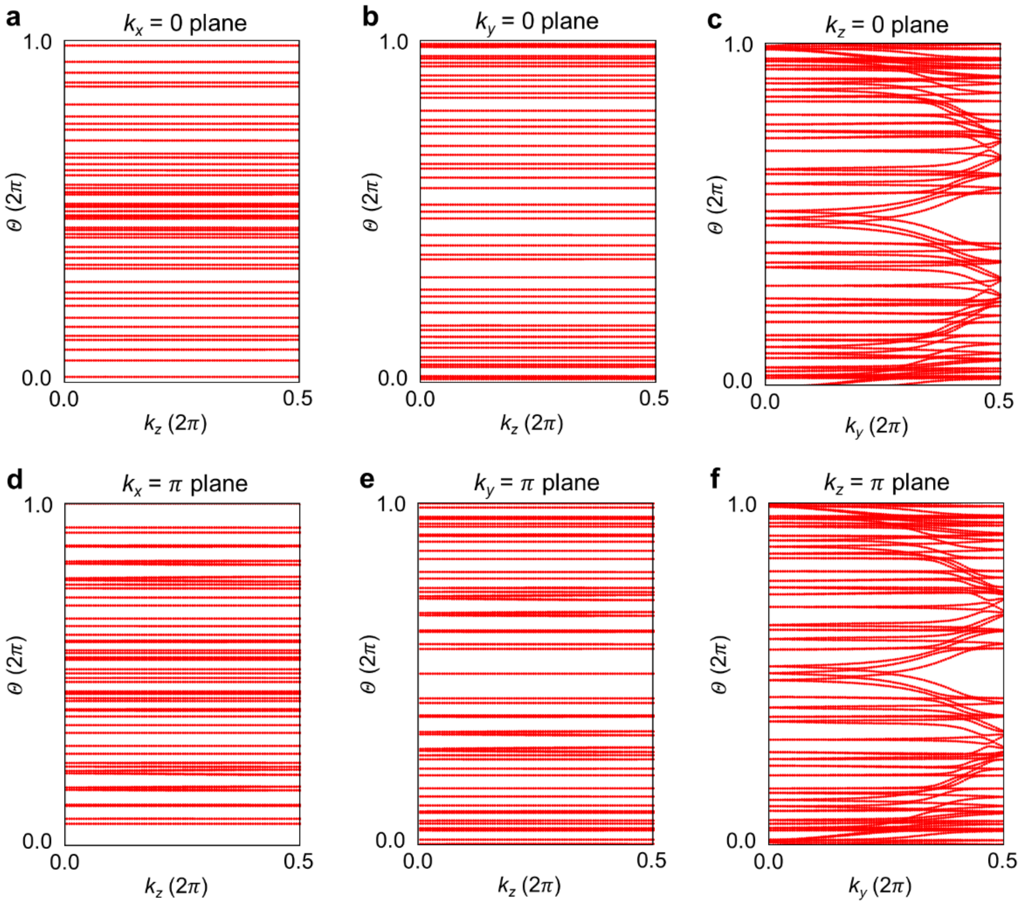

Supplementary Figure 5. Topological property of $Bi_4Br_2I_2$. Evolvement of WCCs in 3D $Bi_4Br_2I_2$ at six planes of $k_x = 0$, $k_y = 0$, $k_z = 0$, $k_x = \pi$, $k_y = \pi$, and $k_z = \pi$, respectively. The corresponding intersecting points with *x*-axis parallel line are 0, 0, 3, 0, 0, 3, respectively, indicating the WTI nature with $\mathbb{Z}_2$ invariant (0; 0 0 1).

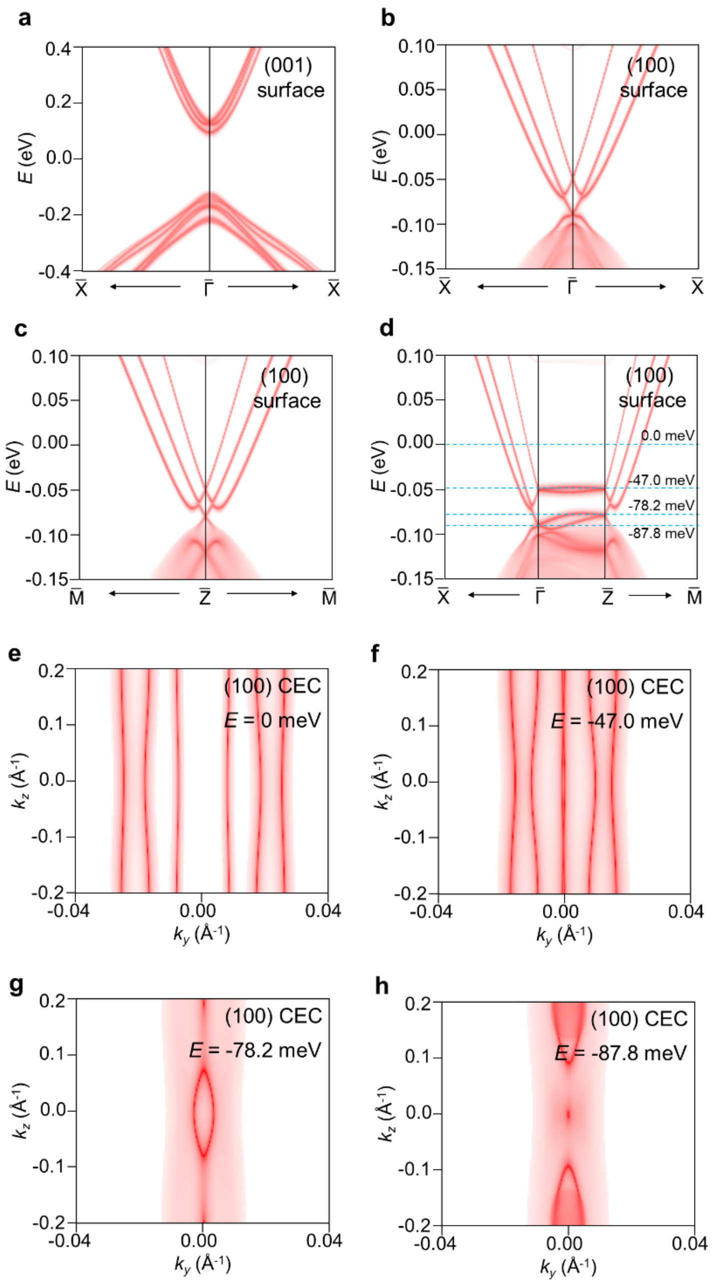

Supplementary Figure 6. Surface bands of $Bi_4Br_2I_2$. a, Surface bands of (001) plane of $Bi_4Br_2I_2$ along $\bar{\Gamma}-\bar{X}$ direction. **b-d**, Surface bands of (100) plane along $\bar{\Gamma}-\bar{X}$, $\bar{Z}-\bar{M}$, and $\bar{X}-\bar{\Gamma}-\bar{Z}-\bar{M}$ paths, respectively. The blue dashed lines in **d** indicate the energy of CEC represented in **e-h**. **e-h**, CEC of (100) plane at different binding energies.

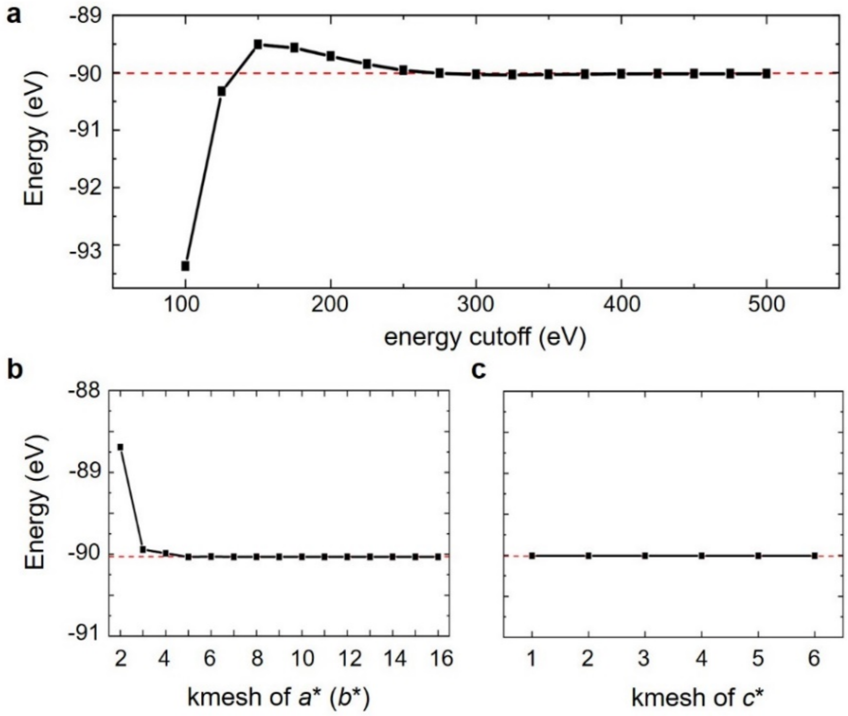

Supplementary Figure 7. Energy convergence tests. a, Energy convergence tests on the energy cutoff. **b**, The number of *k*-grid mesh along the a^* and b^* axes. **c**, The number of *k*-grid mesh along the c^* axis.

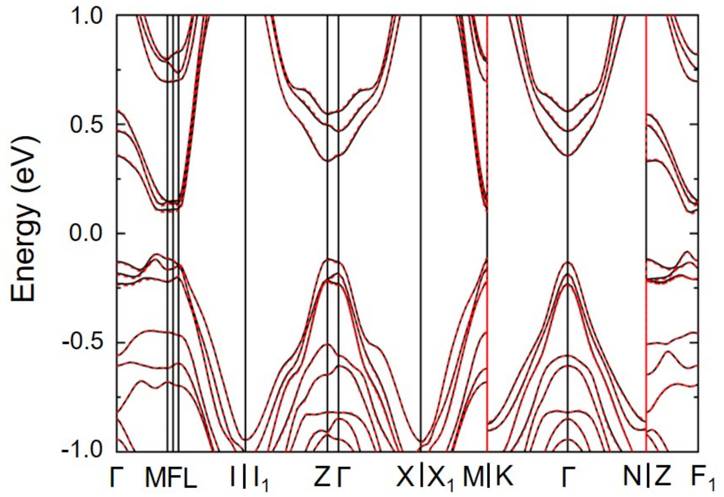

Supplementary Figure 8. Comparison between DFT and TB results. Comparison of bulk band structure calculated from DFT (black solid lines) and MLWF-based TB model (red dashed lines).

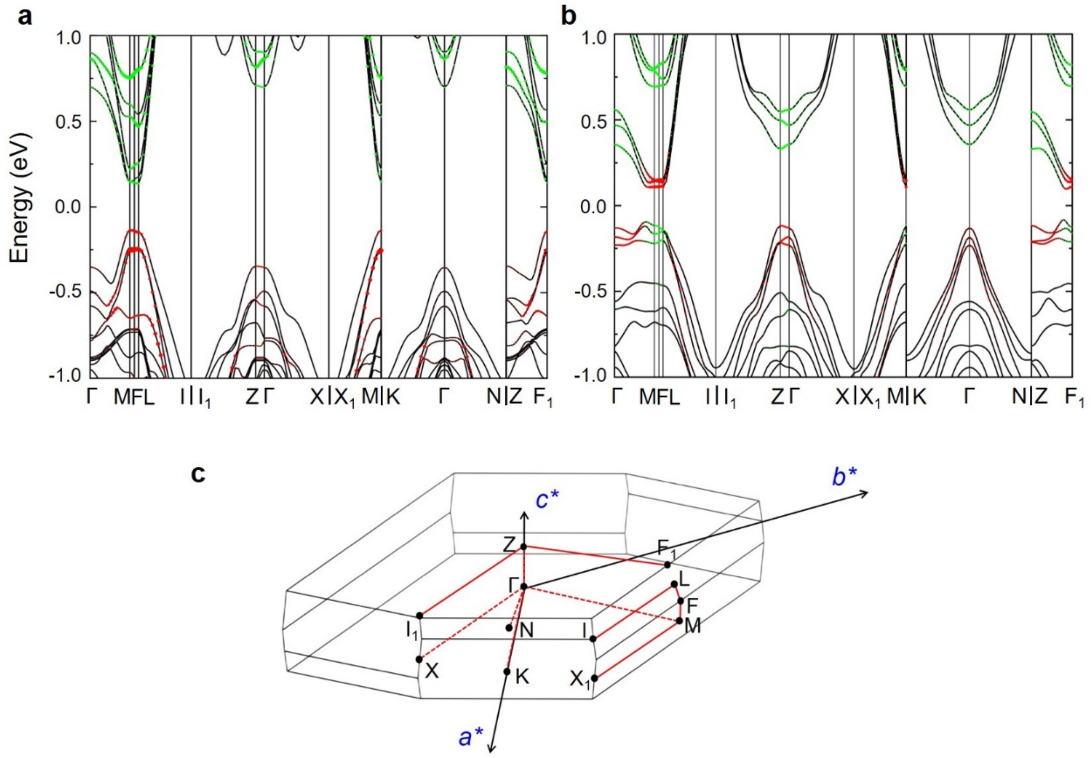

Supplementary Figure 9. Band structure along full high-symmetry lines. a and **b**, Band structures of $Bi_4Br_2I_2$ without (**a**) and with SOC (**b**) along the full high-symmetry lines. The red and green dots represent the weight of p_x orbital of Bi-in and Bi-ex atoms, respectively. **c**, Brillouin zone of primitive cell of $Bi_4Br_2I_2$, where the high-symmetry points and lines are marked by black dots and red lines, respectively.

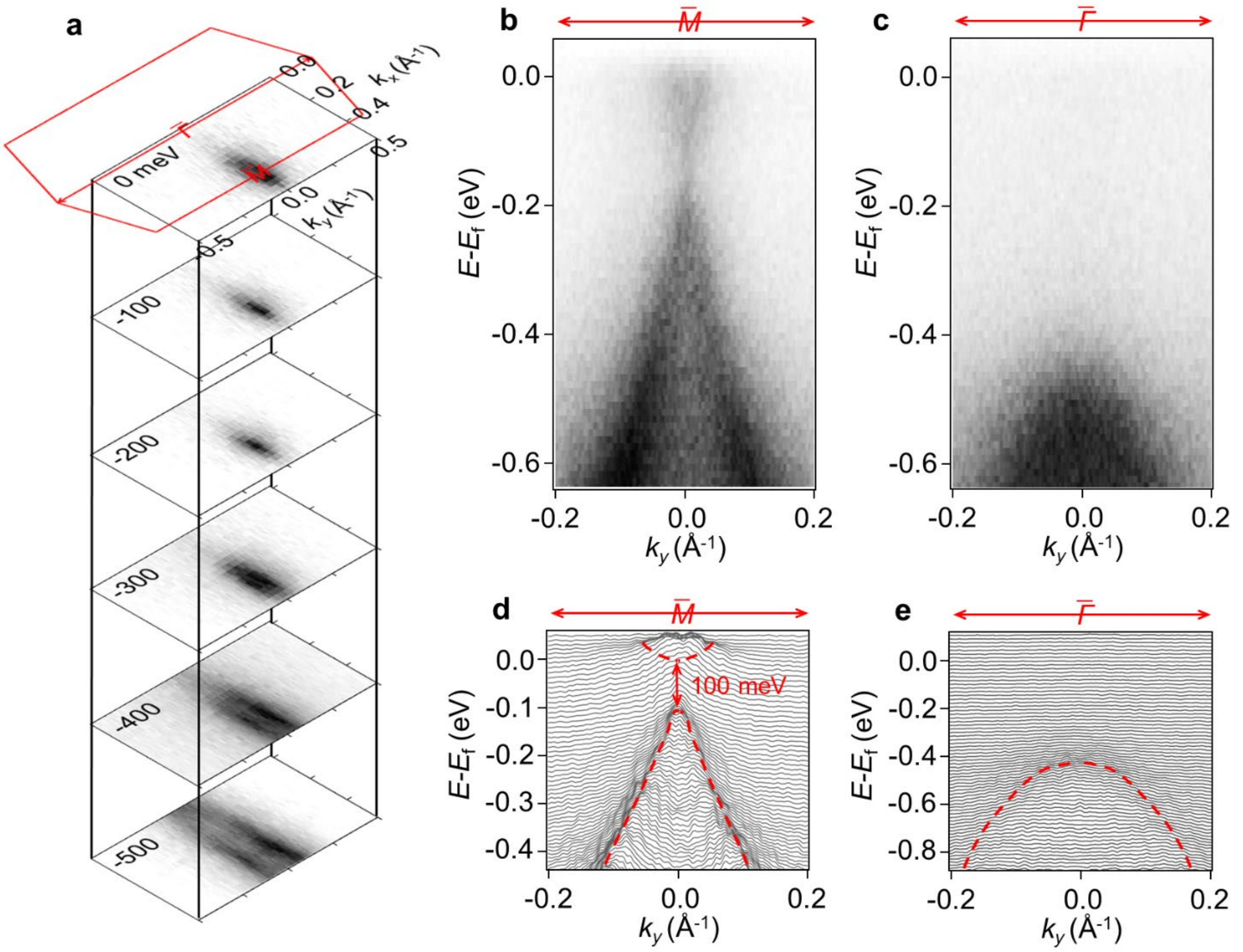

Supplementary Figure 10. Helium light ARPES results. a, CEC at different binding energies of (001) plane $Bi_4Br_2I_2$ measured by Helium light (~21.2 eV). The (001) projected BZ with TRIMs are displayed. **b**, **c**, Energy momentum dispersion along k_y direction at $\bar{M}$ and $\bar{\Gamma}$ points, respectively. **d**, MDC spectra along k_y direction at $\bar{M}$. **c**, MDC spectra along k_y direction at $\bar{\Gamma}$. The red dashed lines are labelled to denote the band dispersion.

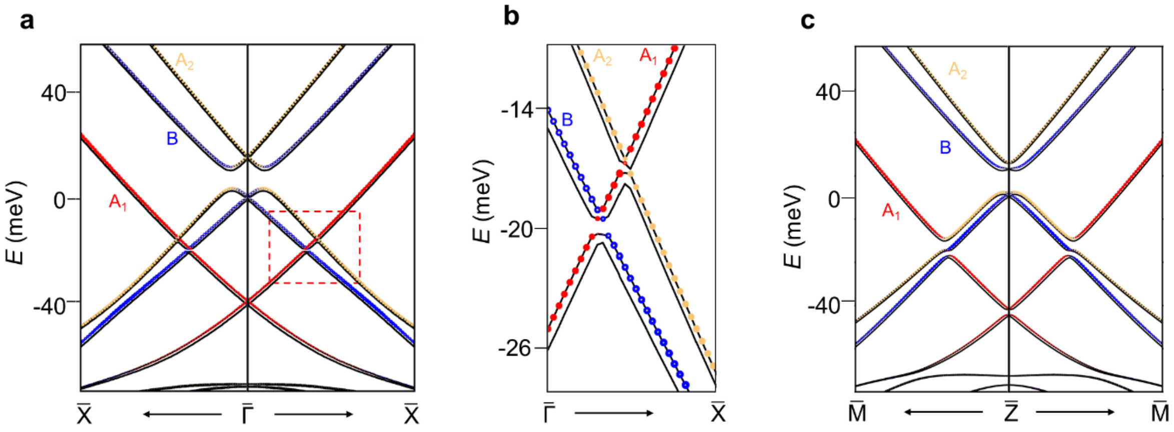

Supplementary Figure 11. Details of topological surface states. a, The topological surface states of $Bi_4Br_2I_2$ (100) surface along $\bar{\Gamma}-\bar{X}$ direction. Three Dirac points at $\bar{\Gamma}$ point and one gapped state away from $\bar{\Gamma}$ can be directly observed. **b,** Enlarged band structure of the dashed square in **a**. There are two finite gaps opened by interlayer coupling between different layers away from $\bar{\Gamma}$ point. **c**, Topological surface states along $\bar{Z}-\bar{M}$ direction with the similar three Dirac points at $\bar{Z}$ and three coupling-induced gaps away from $\bar{Z}$.

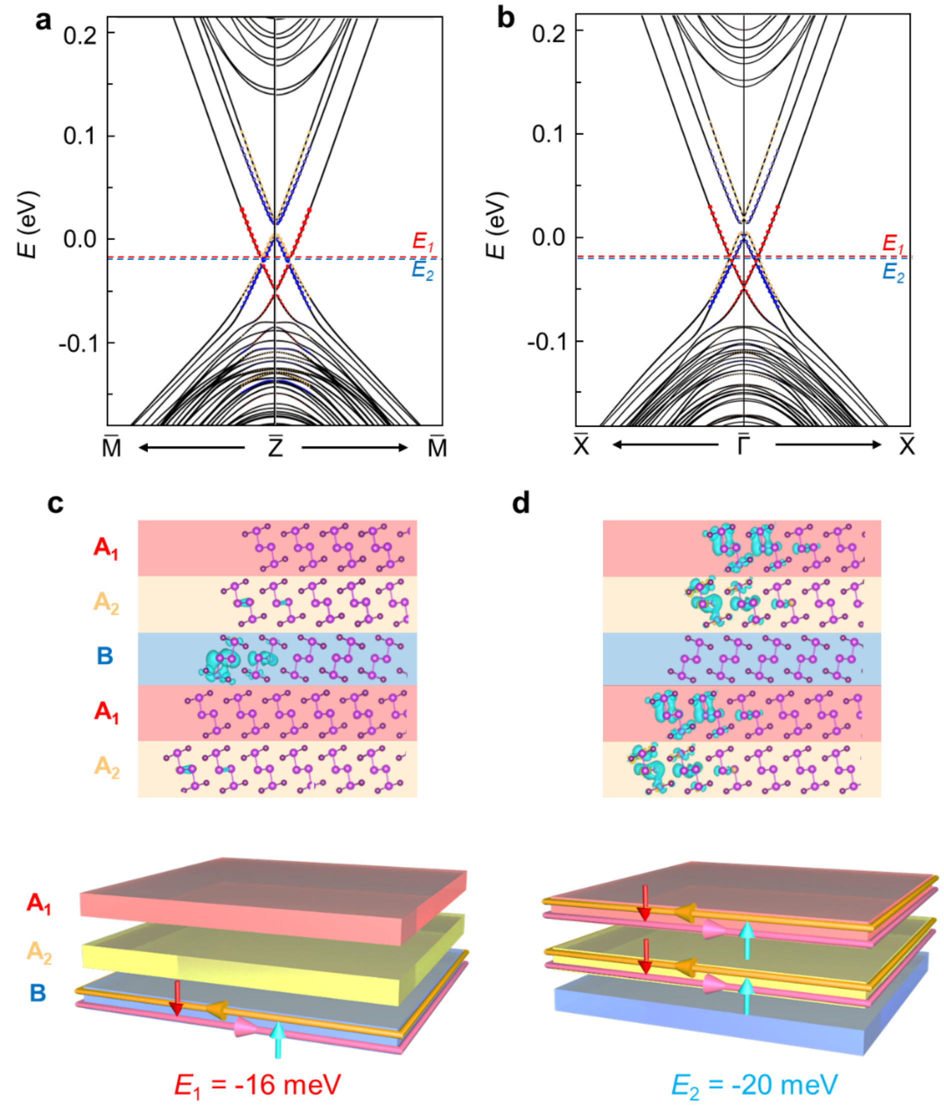

Supplementary Figure 12. Another two conducting modes. a, b, Topological surface states of (100) surface along $\bar{Z}-\bar{M}$ and $\bar{\Gamma}-\bar{X}$ directions, respectively. **c, d**, Corresponding charge distribution when Fermi level locates at E_1 and E_2, respectively.

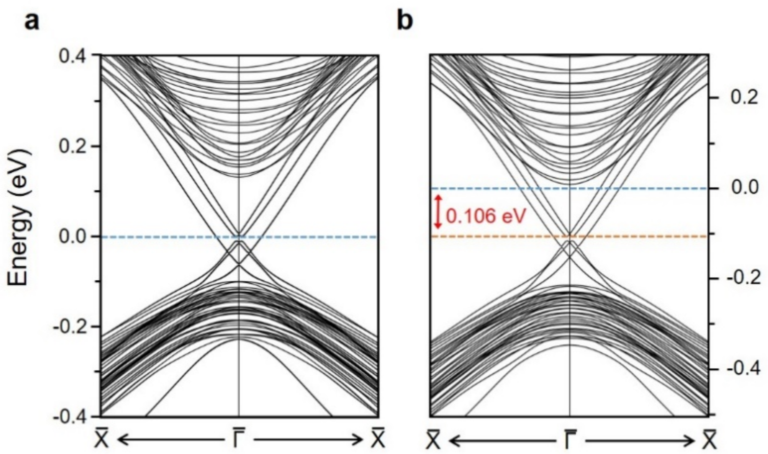

Supplementary Figure 13. Identical Band structure with extra electrons. The (100) surface band structure without additional charges (**a**) and with 0.1 extra electrons (**b**) in the 11-layers-thick slab. The Fermi level is set to zero, as emphasized by blue dashed line.

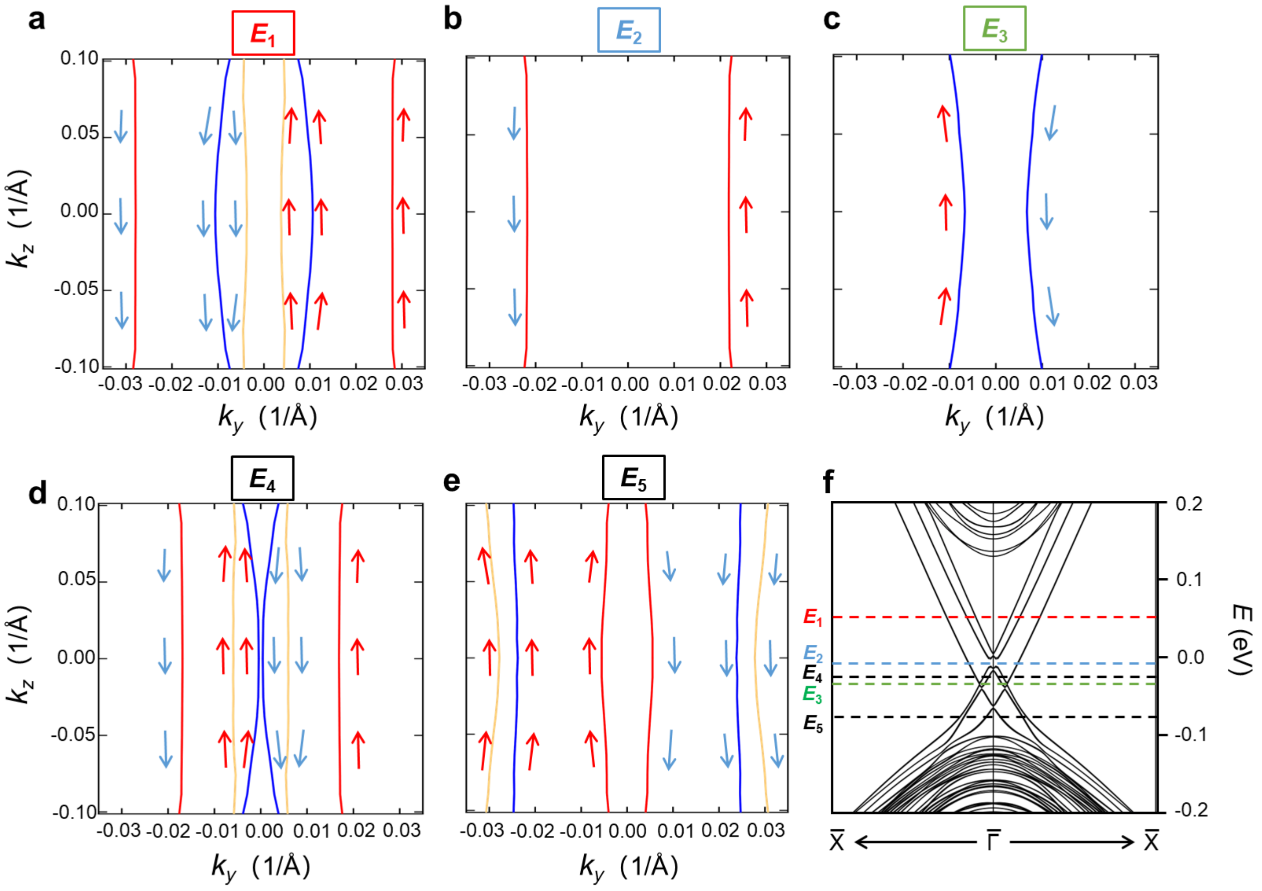

Supplementary Figure 14. Fermi surface at different energies. Fermi surfaces of (100) surface band with five different Fermi energies (**a**-**e**) as depicted in **f**. The selective binding energies of E_1, E_2 and E_3 are the same as those in the main text. The red, yellow and blue colors in **a**-**e** represent the contributions from A_1, A_2 and B layers, respectively. The red and blue arrows depict the spin textures of the surface states.

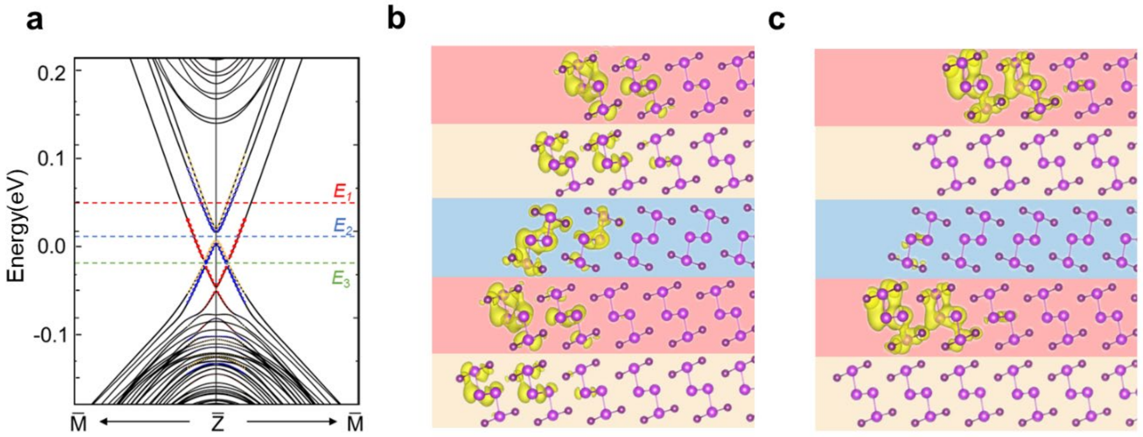

Supplementary Figure 15. Partial charge densities. Surface band structure (**a**) and partial (band decomposed) charge densities in the entire half Brillouin zone of k_y>0 with a very small energy range (~1 meV) around E_1 (**b**) and E_2 (**c**).

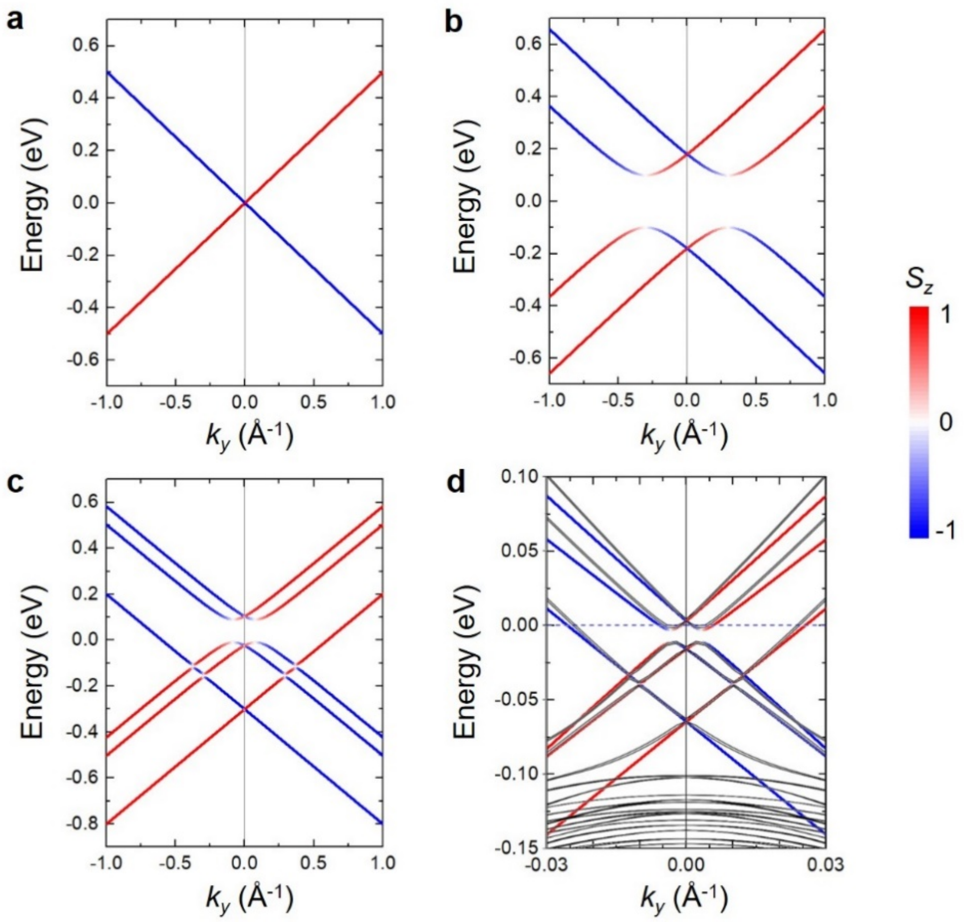

Supplementary Figure 16. Surface Hamiltonian model. a-c, Bands calculated using the surface Hamiltonian models, i.e., Supplementary Equation 2-5. The color from blue to red denotes the eigenvalues of s_z. **d**, Comparison of bands between Hamiltonian model (colored lines) and DFT calculations (black lines). The parameters are: $\hbar v_F = 0.5$ eV·Å in **a**; $\hbar v_{F1} = \hbar v_{F2} = 0.5$ eV·Å, $\varepsilon_1 = -\varepsilon_2 = 0.15$ eV, $m = 0.1$ eV in **b**; $\hbar v_{F1} = \hbar v_{F2} = \hbar v_{F3} = 0.5$ eV·Å, $\varepsilon_1 = 0.08$ eV, $\varepsilon_2 = 0.0$ eV, $\varepsilon_3 = -0.3$ eV, $m_{12} = 0.05$ eV, $m_{13} = m_{23} = 0.008$ eV in **c**; $\hbar v_{F1} = 2.836$ eV·Å, $\hbar v_{F2} = 2.432$ eV·Å, $\hbar v_{F3} = 2.538$ eV·Å, $\varepsilon_1 = 0.002394$ eV, $\varepsilon_2 = -0.01481$ eV, $\varepsilon_3 = -0.06453$ eV, $m_{12} = 0.00406$ eV, $m_{13} = 0.000186$ eV, $m_{23} = 0.000664$ eV in **d**.

Supplementary References

1. Huang, J. *et al*. Room-temperature topological phase transition in quasi-one-dimensional material Bi_4I_4. *Phys. Rev. X* **11**, 031042 (2021).
2. Noguchi, R. *et al*. A weak topological insulator state in quasi-one-dimensional bismuth iodide. *Nature* **566**, 518-522 (2019).
3. Noguchi, R. *et al*. Evidence for a higher-order topological insulator in a three-dimensional material built from van der Waals stacking of bismuth-halide chains. *Nat. Mater.* **20**, 473-479 (2021).
4. Shumiya, N., *et al*. Evidence of a room-temperature quantum spin Hall edge state in a higher-order topological insulator. *Nat. Mater.* **21**, 1111–1115 (2022).
5. Liu, C. C., Zhou, J. J., Yao, Y. & Zhang, F. Weak topological insulators and composite Weyl semimetals: β-Bi_4X_4 (X = Br, I). *Phys. Rev. Lett.* **116**, 066801 (2016).
6. Fu, L., Kane, C. L. & Mele, E. J. Topological Insulators in Three Dimensions. *Phys. Rev. Lett.* **98**, 106803 (2007).
7. Fu, L. & Kane, C. L. Topological insulators with inversion symmetry. *Phys. Rev. B* **76**, 045302 (2007).
8. Hinuma, Y., Pizzi, G., Kumagai, Y., Oba, F., Tanaka, I. Band structure diagram paths based on crystallography. *Comput. Mater. Sci.* **128**, 140-184 (2017).
9. Setyawan, W., Curtarolo, S. High-throughput electronic band structure calculations: Challenges and tools. *Comput. Mater. Sci.* **49**, 299-312 (2010).
10. Kiss, T. *et al.* Quasiparticles and Fermi liquid behaviour in an organic metal. *Nat. Commun.* **3**, 1089 (2012).
11. Otrokov, M. M. *et al.* Prediction and observation of an antiferromagnetic topological insulator. *Nature* **576**, 416-422 (2019).
12. Bellaiche, L. & Vanderbilt, D. Virtual crystal approximation revisited: Application to dielectric and piezoelectric properties of perovskites. *Phys. Rev. B* **61** (12), 7877-7882 (2000).
13. King-Smith, R., & Vanderbilt D. Theory of polarization of crystalline solids. *Phys. Rev. B* **47**, 1651-1654 (1993).
14. Resta, R. Macroscopic polarization in crystalline dielectrics: the geometric phase approach. *Rev. Mod. Phys.* **66**, 899-915 (1994).
15. Eckhardt, C., Hummer, K., Kresse, G. Indirect-to-direct gap transition in strained and unstrained Sn_xGe_{1-x} alloys. *Phys. Rev. B* **89**,165201 (2014).
16. Fadila, M., Nadir, B., El-Houda, F. The elastic constants and related mechanical properties of $Al_xIn_{1-x}P$. *Emerg. Mater. Res.* **9**,1060-1065 (2020).
